# The Foreign Journals: Pathology, Practical Medicine, and Therapeutics

**Published:** 1840-04

**Authors:** 


### PATHOLOGY, PRACTICAL MEDICINE, AND THERAPEUTICS.

### *Aneurism of the Ascending Aorta.* By M. Dubois.

At the meeting of the Royal Academy of Medicine of Paris, 5th Dec., 1839, M. Dubois exhibited an interesting specimen of morbid anatomy, illustrative of the tendency of aneurismal as well as other tumours, to approach the surface, notwithstanding every natural obstacle. The diseased vessel was the ascending aorta, from which, while yet within the pericardium, might be seen a tubular prolongation, of the diameter of two inches, proceeding at right angles directly outwards, and divided near its middle in two unequal parts, by a kind of circular valve. Externally, this tube communicated with a large pouch of condensed cellular tissue, lying beneath the pectoral muscles, and extending into the axilla. Three intercostal spaces had been perforated, not only the intercostal muscles, but one of the ribs having been absorbed as the tumour grew, so that the rough edges of the bone were in contact with the fibrous coagula of the aneurism.

The whistling respiration and œdema of the upper extremities observed during life clearly arose from the pressure of the tumour.

[The foregoing case confirms the truth of the observation that aneurisms of the arch of the aorta more commonly rise towards the sternal surface of the chest. Corvisart, Duverney, Morgagni, and others have given instances of this kind.]

*Bulletin de l'Académie de Médecine.* *Dec.* 31, 1839.

---

### *On the Various Circumstances which appear, in the Course of Diseases, to produce the Curved Form of the Nails.* By M. Vernois.

This paper is sufficient to determine the value, or rather the want of value, of curved finger-nails as a sign of disease. The results, obtained from the examination by the numerical method of 276 cases of various diseases, are as follows:

In any collection of patients, whatever be their diseases, curved nails will be found in at least one third. Among different diseases, phthisis, scrofula, and other chronic affections, have a very marked, though not an absolute or constant influence. Of 88 patients with curved nails, 70 were phthisical or scrofulous; and of 188 with normally formed nails, 40 were suffering from the same conditions. Of the same 88, nine only laboured under acute diseases. Females present this alteration about three times more frequently than males. It is most commonly observed between the ages of ten and thirty. Occupation has no influence upon it. The constitution which coincides with it in five sixths of the cases in which it occurs is that which is marked by a pale, fine, and anæmic skin, blond hair, blue or brown eyes, bluish sclerotica, very long eyelashes, and weak muscles.

*Archives Générales de Médecine. Nov.*, 1839.

---

*Apoplexy of the Eyes.* By Dr. HOLSCHER.

A ROBUST country girl, of eighteen, was hard at work in the harvest field one hot day, and had been frequently stooping to bind up sheaves, when, without any external cause, but with great congestion of blood about the head, she became suddenly blind. When examined a short time after, all sight was lost; the eyes were fixed and felt tense; the conjunctiva was very vascular, there was considerable congestion about the head, and the carotid arteries pulsated strongly. The pupils were widely dilated, and did not in the least contract even when a strong light was brought close to the eye. In the right eye, on viewing it from the side, a slight red tinge of the aqueous humour was perceptible; and with a lens a very small coagulum of blood was discovered lying at the bottom of the anterior chamber. The patient, except for her blindness, was in the same robust health which she had always enjoyed.

Feeling no doubt that the sudden loss of sight was produced by an equally sudden congestion of the eye by blood rushing, as in apoplexy, into the vessels of the choroid and retina, and producing paralysis of the latter, the author ordered a bleeding, to sixteen ounces, from the arm, cold lotions to the head, a saline foot-bath, saline medicines, and an extremely low diet. By a continuance of similar means, with the addition of mercury and a permanent blister at the back of the neck, the patient was so far recovered on the fourth day as to be able to distinguish objects placed before the eyes, and in three weeks she returned to the country with her sight perfectly restored.

*Hannoversche Annalen.* Band ii. Heft. 4.

---

*New Clinical Researches on Sanguineous Concretions formed during life in the Heart and large Vessels.* By Professor BOUILLAUD.

[OF two articles recently published with the above title, the first has no claim thereto, as it is simply an abstract of the opinions of Corvisart and Laennec, and of the author's previous writings on the subject of cardiac concretions. Of the second, in which M. Bouillaud endeavours to prove that the phlegmasiæ in general, and especially pneumonia, exercise a powerful influence on the formation of the coagula in question, the following summary contains the more important particulars. One case of angina tonsillaris, and fourteen of pneumonia, are briefly reported as furnishing the grounds for the doctrine.]

I cannot believe that the large masses of coagula found in the cases just related were wholly formed some days before death, although they possessed the characters assigned to concretions of that date: they were no doubt in part formed in *articulo mortis*, and after death. The mechanism by which concretions of this latter species are produced may be compared with that regulating the coagulation of the blood removed from the vessels during life: in both cases there was a sort of plastic white fibrinous buff, so that these concretions, like the clot, may be termed *buffy*. Be this as it will, it is indubitable that in every case of acute fatal pneumonia, which has occurred in my wards within the last three years, I have found sanguineous concretions, *evidently formed in part before death,* in the heart and blood-vessels; whence I have no hesitation in establishing it as a LAW of pathological anatomy, *that these coagula exist invariably in subjects who die of well-marked acute pneumonia advanced to the second or third degree.* From our knowledge of the notable tendency of the blood to coagulate in direct idiopathic inflammations of the heart and vessels, we might have *à priori* announced that the same phenomenon would occur in the case of the prolonged febrile reaction accompanying inflammation of important organs. For, in truth, what is this febrile reaction, unless a *sympathetic* irritation of the sanguineous system? [Fever is, in the language of M. Bouillaud, merely an *angeio-carditis !*] This is so true, that in some instances we find actual inflammation of the internal membrane of the heart and aorta, instead of a simple sympathetic irritation. In pneumonia, too, the proximity of the inflamed parts to the heart and great vessels must not be forgot, as a condition facilitating the development of inflammation in the latter. [After enumerating the principal signs (none of these are novel) by which he was enabled to diagnosticate with more or less confidence the existence of cardiac concretions in these cases, M. B. continues:] The diagnosis of sanguineous concretions of the heart and great vessels is not a matter of mere curiosity. On the contrary, it is of real importance, in establishing the prognosis of pneumonia, to ascertain whether or not the complication in question exists. This complication seriously increases the patient's danger, when the concretions are abundant, and form a considerable obstacle to the free circulation of the blood through the cavities of the heart and great vessels. [But a moment since the writer laid it down as a LAW, that these concretions invariably exist in the second and third stages of pneumonia; he now bases his favorable prognosis in inflammation of the lungs on their *absence.*] In a therapeutical point of view also, this is an important consideration. It is not very unusual to find the pulse embarrassed, weak, and small, even when the patient himself is strong, the inflammation intense and extensive, and the heart's pulsations energetic. Under these circumstances, the majority of practitioners recommend us not to bleed; they consider the smallness of the pulse as an infallible sign of radical debility. But the truth is, that the phenomenon in question depends on the presence of coagula in the heart; bleeding is specially indicated for its removal, and, as I have ascertained in numerous instances, restores the pulse to its accustomed fulness, and raises, instead of depressing, the patient's strength.

*L'Expérience*, Nos. xcvi. and c., *Mai*, 1839.

---

*On a Contagious Growth of Conferva on the Water Salamander (Triton punctatus).* By Dr. A. HANNOVER, of Copenhagen.

THIS disease was first accidentally observed in the summer of 1838 on a dead salamander, which Dr. H. having dissected had put back into the same vessel with several living ones. After some days he found it covered with a confervoid efflorescence, which, as seen through the water, looked like mucus. Some of the living salamanders in the same vessel had had the tips of their tails cut, to observe the process of their reproduction; in some the tail had been quite removed, and in others the spinal cord only had been divided. The same efflorescence soon appeared in the wounds of all that had been thus operated on. It always began growing on the surface of the wound, and thence spread forward on the sides of the remains of the tail, giving all the parts that it attacked a dark colour. If it reached the anal aperture the animal always died. Sometimes, however, the cuticle separated and the efflorescence fell off with it, and the animal recovered. At first also it could be scraped off with the cuticle; the subjacent skin was then usually found smooth, but of a dark colour from commencing gangrene. In this case, about sixteen hours after, the efflorescence would have again sprouted more thickly than before, and could not now be scraped off, probably because it had struck root in the cutis. In this short time it would reach the height of half a line, and in hours more of a line; sixteen hours later it would be half an inch higher, it would reach the anal orifice, and the animal would die.

Even a slight injury, such as the prick of a needle, was followed by the same phenomena, and in some cases the efflorescence appeared without a wound. The toes were in several cases thus affected without previous injury, the efflorescence shooting out in a tuft from them, and after a time falling off with all that it had infested, so as to maim one or more of the extremities. The same animal was liable to be thus affected several times.

The plant that produces this disease is a conferva of a similar kind to that which commonly grows on dead flies. It grows very rapidly, often half an inch in four or five days; then it ceases to increase in length, but the transparency which it had at first gradually changes to a whitish colour, and its filaments become covered with buds. The filaments are simple or very rarely branched membranous tubules containing a granular substance. They are of varied diameter, and pointed or sometimes swollen at their ends, and they are often partitioned into cells. When quite ripe the granular matter leaves the interior of the tubes and adheres to their outer surfaces.

Having established these observations, and meeting with M. Audouin's work on the Muscardine, Dr. Hannover determined on testing the contagious nature of the plant by inoculating unripe confervæ into the back of a healthy salamander. After sixteen hours they had grown and were a line high; the animal was severely affected, and swam with his back bent forwards, but he generally remained quiet at the bottom of the water. Sixteen hours after the efflorescence had extended over the whole wound, and was a line and a half high; in sixteen hours more it was two lines high, and its ends were covered with buds. The animal was now about to cast his skin, and after sixty-four hours it fell off with the efflorescence and left a perfectly smooth surface. The wound appeared healthy though not reduced in size, and as soon as the efflorescence was removed the animal became unusually lively.

Three days after, the same salamander was again inoculated in the same place and again recovered after casting his cuticle. A third time he was inoculated with ripe confervæ, and again recovered after presenting the same series of phenomena.

A very lean and weak salamander was similarly inoculated. In twenty-four hours the conferva had grown a line high, and he died shortly after. Another of the same kind was inoculated with conferva from a dead fly; he soon after exhibited signs of severe pain, turned on his side, and struck out violently with his tail; but after casting his cuticle he recovered in three days.

*Müller's Archiv.* Heft iv., 1839.

---

*Remarkable Case of Ischuria Renalis, of Nine Years' Standing, with Vicarious Vomiting of Urine.* By F. L. KREYSIG, M.D., Dresden.

S. B., æt. twenty-five, six years after the comencement of menstruation, began to complain of abdominal pain, general spasm, and difficulty in voiding urine and fæces, succeeded by orthopnœa, and symptoms of thoracic and abdominal inflammation, which were treated antiphlogistically. The bladder required the frequent use of the catheter. The patient suffered pain in the right knee, and was obliged to keep it drawn upwards towards the abdomen. Two years after, the legs began to swell, respiration became still more difficult, the urinary secretion *ceased,* and the patient vomited a fluid containing, according to chemical analysis, the principles of urine.

M. Kreysig now detected, in the right iliac region and near the spine, a tumour extending towards the liver, exquisitely painful to the slightest touch, and which the patient had noticed for four years. The bowels were invariably costive; and twice a day (five, a.m., and six, p.m.) urinous vomitings occurred, preceded by dyspnœa, so urgent as twice to require v.s. No urine was found in the bladder. Tepid baths, and mercurial and stimulating liniments to the region of the kidney, were recommended. During the time the patient was in the baths, the skin exhaled a very fœtid odour. The tumour gradually enlarged, and grew softer.

The patient now returned home, and four months after (January 24, 1835,) informed M. Kreysig that the tumour had daily increased, become harder and more painful, and pointed in the epigastric region. Fluctuation was now evident, and in three weeks the abscess broke, during a violent spasm, discharging a large quantity of pus, which was soon after succeeded by an increase of suffering. In about six weeks, the urinous vomitings ceased, and urine was voided by the bladder. Shortly afterwards, the pain being then more than usually intense, with an urgent desire to evacuate the bowels, the patient expelled, per anum, a mass about as large as a goose's egg, resembling fat mixed with pus, and intolerably fœtid. From this period, all abdominal swelling and pain ceased, and menstruation returned. In about three-quarters of a year, the health was entirely restored, not one of the former symptoms remaining.

*Hufeland's Journal. July*, 1839.

---

*On the Use of Hydrochlorate of Baryta in Strumous Ophthalmia.*
By Dr. Payan, of Aix in Provence.

Dr. P. having observed that this remedy was used with excellent effect by Lisfranc in scrofulous diseases, he resolved to try it in an obstinate case of ophthalmia accompanied by a high degree of photophobia. The patient was six years of age. He dissolved two grains of the hydrochlorate in three ounces and a half of "eau sucrée," and this quantity was taken in portions during the course of the day. No particular effect being produced, on the third day three grains were taken, and the dose was gradually raised to ten grains in the course of the day. On the twentieth day the medicine was discontinued, the patient being considered nearly well. In another case, twelve grains in the course of the day were taken with excellent effect, and without any symptoms of gastric irritation being produced. During the administration of the remedy, Dr. Payan orders a light and sparing diet, considering that harm is frequently done by the tonic medicines and stimulating regimen which are generally ordered as a matter of course in strumous ophthalmia. It is true that the scrofulous constitution is often accompanied by an atony which is marked by a pale and pasty complexion, feeble circulation, a low degree of sensibility, and a general indolence of mind and body; but the same scrofulous disposition is frequently developed in persons of a lively temperament, animated countenance, habitually quickened circulation, and mobile and easily excitable nervous system. It is in this latter class of persons that we most frequently find that acute sensibility of the retina which produces photophobia, with the accompanying spasmodic contraction of the eyelids, and abundant secretion of tears. In these cases, a generous diet with wine and tonics will increase the general excitement, and consequent irritability of the retina, whilst the barium appears to act as a sedative, and combined with a mild diet produces a general soothing effect. *Revue Médicale. April*, 1839.

---

*On the Treatment of Dysentery with Albumen.* By M. Saucerotte,
Physician to the Hospital at Lunéville.

The dysentery which annually attacks a considerable number of men of our garrison, affected only thirteen in the months of October and November, 1838, and ten in those of August and September, 1839. I employed in some of these cases the albuminous method of treatment recommended by MM. Bodin de la

Pichonnerie and Mondière. Comparatively with those by the ordinary method (by antiphlogistics and opiates), the results obtained by it were really of the most striking kind. My own experience and that acquired by others in the hospital shows that when the disease is at all severe, it generally lasts two or three weeks, sometimes becomes chronic, or terminates in death: besides this the reiterated application of leeches causes severe pain, the administration of enemata is continually attended with much difficulty, the convalescence is prolonged, and attended with extreme weakness when the disease is not stopped during the first week. Judging from the cases I have observed, the albuminous treatment is attended with precisely contrary effects—rapid cessation of the symptoms, quick convalescence, and recovery of strength, while the administration is attended neither with pain nor any sort of inconvenience.

[The albumen was exhibited both as a ptisan and in enemata, according to the formula of M. Mondière, and eight cases are related (some, however, with ludicrous brevity) in support of the above emphatic encomiums. In some of the cases there was undoubted inflammation of the large intestine; in all frequent bloody stools, tenesmus, &c.]

*Gaz. Médicale de Paris.* No. xlvii. *Nov.* 1839.

---

*On the Employment of the Oil of Cod Fish in Scrofulous Diseases.*
By M. TAUFFLIED.

[THE beneficial influence of the oil of cod fish in certain forms of scrofulous disease, has within the last few years been dwelt on by various German writers; M. Taufflied reports eight cases from which he draws the following inferences confirmative of their accounts:] 1. The oil of cod fish exercises a favorable influence on the general state of lymphatic subjects. 2. If administered with proper care it possesses the property of curing scrofula of the bones, tabes mesenterica, and scrofulous or rheumatismal chronic arthritis. 3. Caries accompanied with solution of continuity and engorgement of the soft parts requires local treatment in addition to the oil administered internally. Under these circumstances compression and alcoholic ioduretted fomentations may be successfully employed. 4. The oil of cod fish has no efficacy in cases of gouty arthritis, nor does it exercise any influence on the engorgement of any lymphatic glands except the abdominal. It seems to have little or no effect on scrofulous phthisis, if this be at all advanced. 5. The oil should be administered perseveringly and for several months in order to secure advantageous results.

*Gaz. Médicale de Paris.* No. xlv. *Nov.* 1839.

